# Ultrasonography demonstrates vagus nerve atrophy in Parkinson’s disease: a meta-analysis

**DOI:** 10.1007/s00415-026-13993-w

**Published:** 2026-07-15

**Authors:** Laura C. J. Sijben, Sander M. J. van Kuijk, Ali Jahanshahi, Werner H. Mess, Marcus L. F. Janssen

**Affiliations:** 1 Department of Clinical Neurophysiology, Maastricht University Medical Center, P. Debyelaan 25, 6229 HX Maastricht, the Netherlands; 2 Mental Health and Neuroscience Research Institute, Faculty of Health, Medicine and Life Sciences, Maastricht University, Universiteitssingel 40, 6229 ER Maastricht, the Netherlands; 3 Department of Neurology, Maastricht University Medical Center, P. Debyelaan 25, 6229 HX Maastricht, the Netherlands; 4 Department of Clinical Epidemiology and Medical Technology Assessment, Maastricht University Medical Center, P. Debyelaan 25, 6229 HX Maastricht, Netherlands; 5 Department of Neurosurgery, Maastricht University Medical Center, P. Debyelaan 25, 6229 HX Maastricht, the Netherlands

**Keywords:** Parkinson’s disease, Vagus nerve, Ultrasound, Meta-analysis, Autonomic dysfunction

## Abstract

**Background:**

Parkinson’s disease (PD) is a progressive neurodegenerative disorder characterized by motor and non-motor symptoms, including autonomic dysfunction. Increasing evidence suggests that PD pathology may originate in the enteric nervous system and propagate to the brain via the vagus nerve. Accordingly, structural alterations of the vagus nerve could reflect disease-related neurodegeneration and may serve as a biomarker for PD. Vagus nerve ultrasound enables non-invasive measurement of the vagus nerve by measuring the cross-sectional area (CSA). However, existing studies report conflicting findings regarding vagus nerve CSA in PD. This meta-analysis evaluates whether vagus nerve CSA is decreased in persons with PD compared to controls.

**Methods:**

Online databases were searched up to January, 2026, for studies measuring the CSA of the vagus nerve in PD patients using ultrasound. Twelve studies with a total of 1091 participants (541 PD and 550 controls) were included. Inclusion criteria encompassed peer-reviewed studies with PD patients and controls. Quality assessment was conducted using the Newcastle–Ottawa Scale. Meta-analysis was performed using a random effects model accounting for between-study heterogeneity.

**Results:**

This meta-analysis showed a statistically significant reduction in CSA of PD patients compared to the controls. The mean CSA of the right and left vagus nerve was 1.97 mm^2^ and 1.70 mm^2^ in PD patients and, 2.21 mm^2^ and 1.89 mm^2^ in controls. Study quality was moderate to high but high heterogeneity was observed.

**Conclusions:**

On a group level, there is a reduction of CSA of the vagus nerve in PD patients measured by ultrasound.

**Supplementary Information:**

The online version contains supplementary material available at https://doi.org/10.1007/s00415-026-13993-w.

## Introduction

Parkinson’s disease (PD) is a debilitating neurodegenerative disorder that affects millions of people worldwide [1]. It is characterized by the progressive loss of dopaminergic neurons in the substantia nigra and by the accumulation of α-synuclein-rich Lewy bodies and Lewy neurites [2, 3]. The lack of dopamine results in motor symptoms such as bradykinesia, rigidity and tremor. PD patients not only suffer from motor disabilities, but also experience autonomic symptoms. The vagus nerve plays a major role in regulating many autonomous bodily functions, including digestion, heart rate, and breathing. Moreover, it is suggested that the vagus nerve is one of the major routes that spread α-synuclein from the enteric nervous system to the lower brainstem, i.e., along the gut-brain axis [4–6]. Altogether, a growing body of evidence suggests that the vagus nerve plays an important role in PD [7]. Based on these observations, it has been suggested that the nucleus of the vagus nerve may also degenerates, resulting in a reduction of nerve fibers in the vagus nerve of patients with PD. The degeneration could potentially be linked to the presence of autonomic symptoms.

In recent years, several studies measure the cross-sectional area (CSA) of the vagus nerve in PD patients, using ultrasound as a reflection of neuronal degeneration as a possible diagnostic tool or biomarker [8–20]. Ultrasound is a non-invasive and safe method for imaging the vagus nerve in the neck region. A diagnostic tool can easily be used at the bedside. Therefore, there is a growing interest in using vagus nerve ultrasound to study degeneration of the vagus nerve in PD. However, published findings are inconsistent, with contradictory results regarding the reduction of the CSA of the vagus nerve in PD. Therefore, the aim of this study is to conduct a meta-analysis of the available evidence.

## Materials and methods

This review was reported in accordance with the PRISMA reporting guideline.

### Search

Online databases are searched: Medline (through PubMed), Scopus, and Web of Science up to 1st of January 2026. The search strategy consists of controlled terms and text words for the concepts of Parkinson’s disease, vagus nerve, and ultrasound. The specific searches for each database can be found in the supplementary file.

### Study selection

**Fig. 1:**
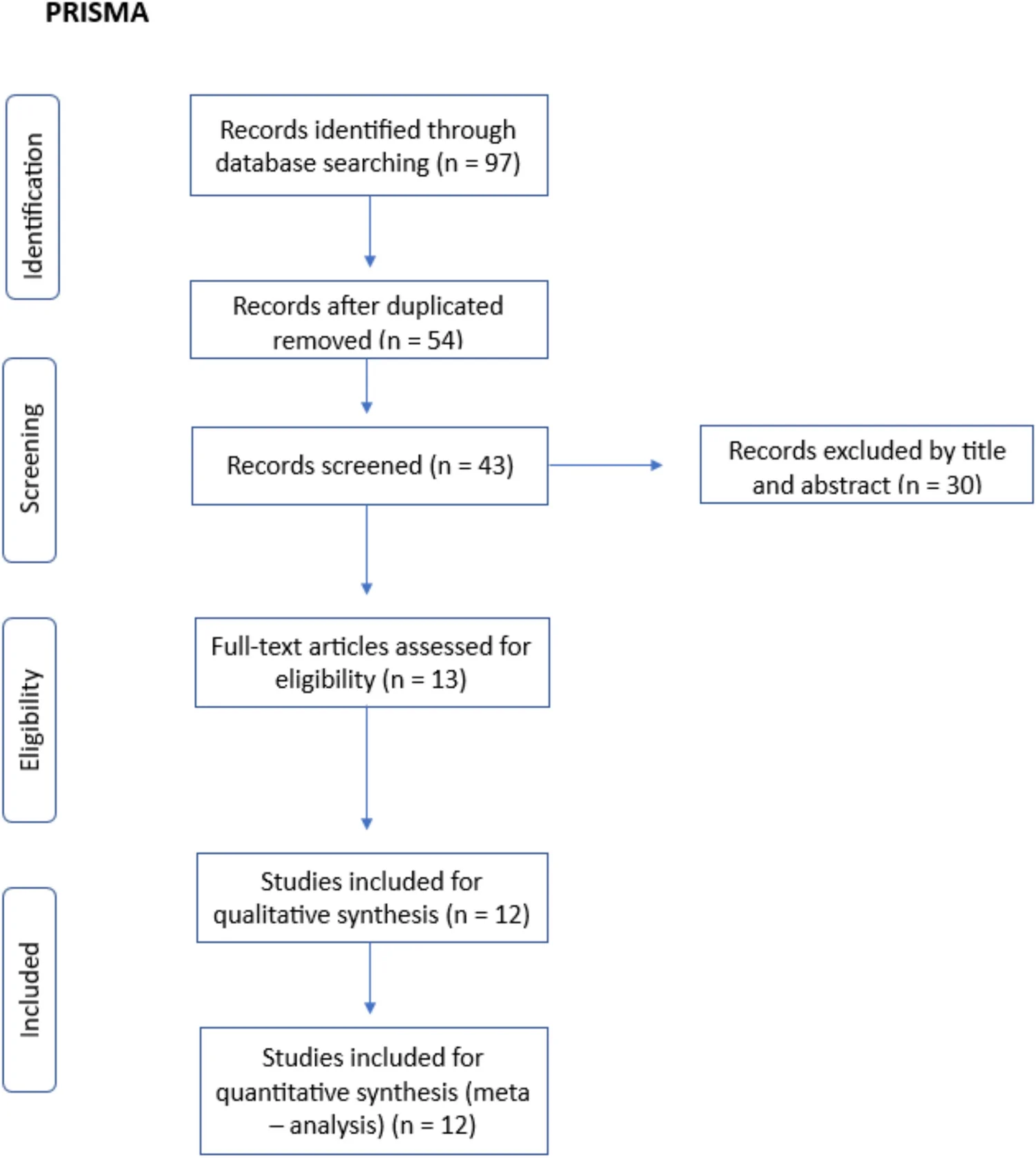
PRISMA flowchart detailing the search process and studies included

Studies that measure the CSA of the vagus nerve by ultrasound in PD patients and a control group are included. Manuscripts have to be written in English, German, or French and be published in a peer-reviewed journal. Sufficient data for qualitative and quantitative analysis need to be available. Studies that measure CSA by any method other than ultrasound or in subjects with diseases other than PD are excluded. We also exclude meta-analyses, systematic literature reviews, and animal studies, although the former are used to screen for missed studies. Finally, we exclude the study by Sartucci et al. from further analysis due to deviating values (> 3 SD) of the CSA of the vagus nerve in the healthy control group. The details of the search strategy and study selection are shown in the following PRISMA diagram (Fig. 1).

### Quality assessment

Two review authors (LS, MJ) independently assessed the risk of bias for each eligible nonrandomized study using the Newcastle–Ottawa Scale (NOS), as described in the Cochrane Handbook for Systematic Reviews of Interventions (Higgins 2011). The NOS is a tool for quality assessment of case–control studies. It consists of a ‘star system’, in which a study is judged on three perspectives, including the selection of the study groups, the comparability between groups, and the ascertainment of outcomes of interest [21]. Studies are categorized into low (0–3), moderate (4–6), or high quality (7–9).

### Data analysis

Quantitative synthesis is performed with meta-analysis of study-level data using a random effects model due to anticipated between-study heterogeneity. In addition, as measurement devices may differ substantially between studies, we compute standardized mean differences and subsequently pool the results. Between-study heterogeneity is expressed as I-squared, which can range between 0 and 100%. Mean and standard deviation are presented, unless indicated otherwise. The study from Tsukita et al. (2018) reports median and interquartile range data, which are converted to mean and standard deviation.

## Results

We include 12 studies [8–16, 18–20] with a total of 1091 participants (541 PD and 550 controls). An overview is provided below (see Table 1). All included studies provided the CSA values (mm2) of both the right and left vagus nerve for PD patients and controls.

**Table 1:** Details of the included studies are given, including year of publication, ultrasound device, side of measurement, number of participants (Parkinson’s disease and control), MDS-UPDRS III score, disease duration Hoehn and Yahr score.

Name	Year	Ultrasound details	Probe frequency (MHz)	Side of measurement	Number of participants		MDS-UPDRS-III (mean, SD)	Disease duration (years)	H&Y I/II-III/IV
					PD	Controls			
Fedkte et al.	2018	Toshive, Aplio 440	15	N/A	32	30 (15/15)	33 (12)	N/A	3/63/34
Pelz et al.	2018	Esaote, MyLab Five (LA435)	15	Thyroid gland	35	35	23 (10) on	10.6 (2.7)	2.7 (1.0)
Tsukita et al.	2018	GE, LOGIQ S8/LOGIC e Expert	12	Distal end common carotid artery	21	21	19 (13–23)	N/A	10/80/10
Walter et al.	2018	Esaote, MyLab Twice (LA435)	15	Thyroid gland	20	61	31 (12)	10 (7)	N/A
Horsager et al.	2021	Philips, EPIQ 7 (L12-3)	12	Thyroid gland	63	56	23 (10)	8 (2–85) months	22/68/10
Sartucci et al.	2021	Esaote, Mylab Gamma	8–19	N/A	20	20	N/A	10 (8)	2.5 (1.0)
Chechetkin et al.	2021	Philips, IU22 (L17-5)	5–17	Distal end common carotid artery	32	32	37 (16)	3 (2–8)	25/75/0
Sijben et al.	2022	Philips, IU22 (L17-5)	5–17	N/A	31	51	N/A	95 (66) months	13/84/13
Ömer Özçağlayan et al.	2022	Toshiba Applio XG	6–14	Thyroid gland	43	44	22 (16)	7 (4)	N/A
Huckemann et al.	2023	Philips, Affinity 70G	18	Level of the bulbus caroticus	80	40	30 (16)	6 (6)	2.7 (0.9)
Höppner et al.	2023	Philips, Affiniti 50/70 (EL 18–4)	5–18	Thyroid gland	49	24	21 (13)	77 (79) months	2.9 (0.5)
Dong et al.	2024	GE LOGIQ E20, United States	4–10	Thyroid cartilage	75	96	*	*	*
Laucius et al.	2025	Philips, EPIQ 7	4–18	Carotid bulb + common carotid after bifurcation	60	60	N/A	N/A	5/10/30/23.3

Values are presented as means + SD

### Quality assessment

The overall quality of studies is moderate to high. Information relating to the quality of each individual study using the Newcastle–Ottawa Scale is provided in Table 2.

**Table 2:** Quality assessment using the Newcastle–Ottawa Quality Assessment Scale

Name	Year	Selection				Comparability	Exposure			Total score
		Q1	Q2	Q3	Q4	Q1	Q1	Q2	Q3	
Fedkte	2018	*	–	–	*	**	–	*	*	6
Pelz	2018	*	–	–	*	**	*	*	*	7
Tsukita	2018	*	–	–	*	**	–	*	*	6
Walter	2018	*	–	–	*	**	*	*	*	7
Horsager	2021	*	–	–	*	**	–	*	*	6
Sartucci	2021	*	–	–	*	**	*	*	*	7
Chechetkin	2021	*	–	–	*	**	–	*	*	6
Sijben	2022	*	–	–	*	**	–	*	*	6
Ömer Özçağlayan	2022	*	–	–	*	**	*	*	*	7
Huckemann	2023	*	–	–	*	**	–	*	*	6
Höppner	2023	*	–	–	*	**	–	*	*	6
Dong	2024	*	–	–	*	**	*	*	*	7
Laucius	2025	*	–	–	*	**	–	*	*	6

A study is awarded a maximum of one star for each numbered item within the Selection and Exposure categories

A maximum of two stars can be given for comparability. Total score is sum of stars

Studies are categorized by score into low quality (0–3), moderate quality (4–6), or high quality (7–9)

### Random effects model

**Fig. 2:**
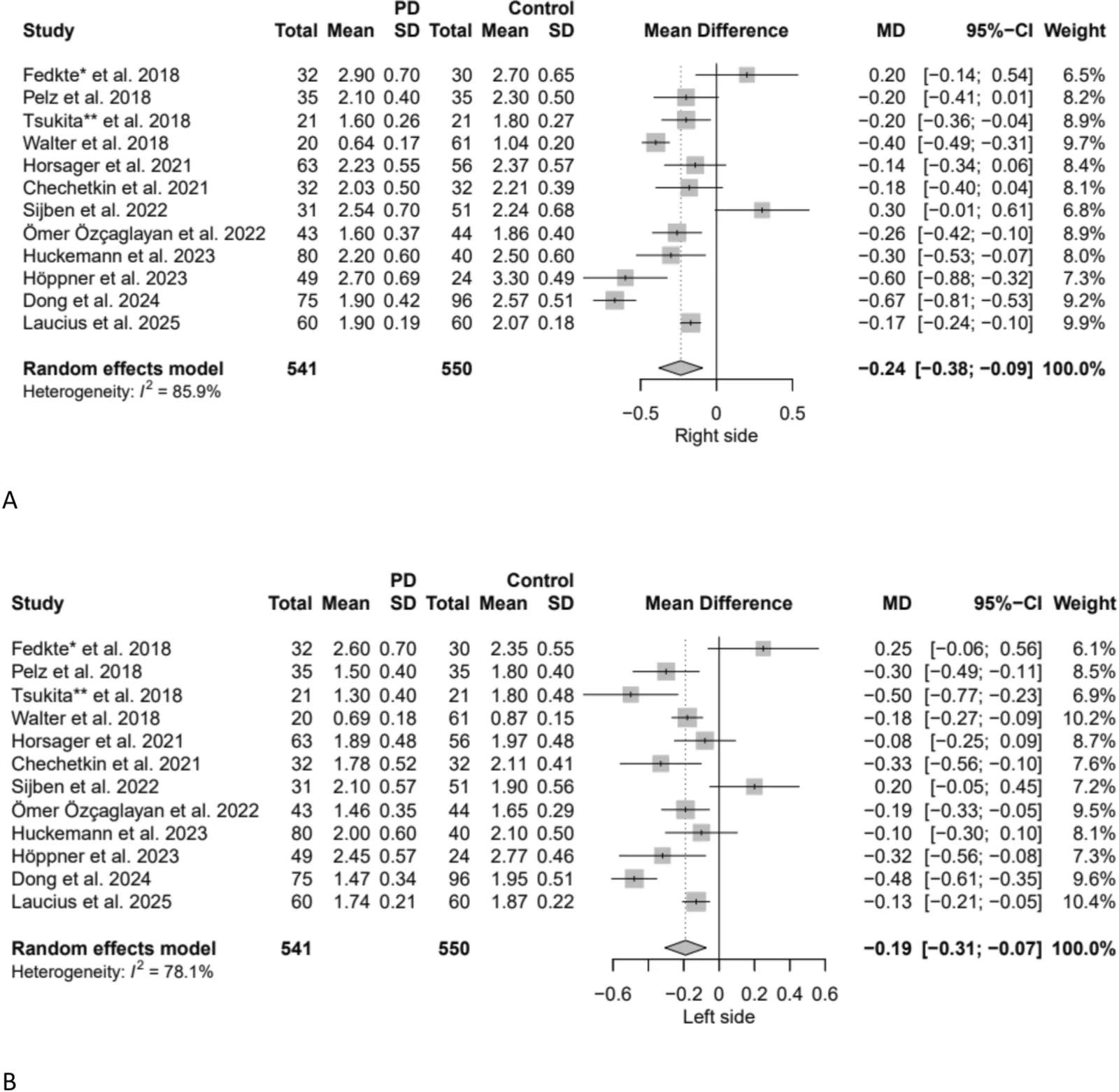
Forest plot of **A** right and **B** left vagus nerve cross-sectional area in Parkinson’s patients vs. controls

The CSA of the right and left vagus nerve in PD patients is 1.97 (SD 0.44) mm^2^ and 1.70 (SD 0.44) mm^2^, respectively, and in the controls 2.21 (SD 0.44) mm^2^ and 1.89 (SD 0.42) mm^2^. The results of our meta-analysis show that the CSA of the vagus nerve is smaller in PD patients by 0.24 mm^2^ (95%CI − 0.38 to − 0.09) and 0.19 mm^2^ (95%CI − 0.31 to − 0.07), for the right and left, respectively. The overall effect is significant for both measurements. It is worth noting that there is substantial heterogeneity between studies (I^2^ = 85.9% and I^2^ = 78.1%) in the right and left CSA of the vagus nerve, respectively. This may be due to differences in study design, sample size, patient characteristics, and ultrasound techniques (Fig. 2).

## Discussion

This meta-analysis of 12 studies shows that the CSA of the vagus nerve is reduced in PD. The observed group difference indicates an association at the population level. Yet, the large within-group variability leads to a substantial overlap of the CSA values in PD patients and controls. As a consequence, vagus nerve ultrasound has limited discriminative ability at the individual patient level.

### Biological mechanisms and clinical implications

The findings of this meta-analysis indirectly confirm that axonal degeneration of the vagus nerve occurs in PD, suggesting atrophy of the vagus nerve plausibly induced by α-synuclein. If α-synuclein pathology propagates via the vagus nerve, it leads to progressive loss of cell bodies, resulting in axonal degeneration and thereby nerve atrophy. A measurable reduction in the CSA of the vagus nerve supports the existence of distinct PD subtypes. In recent years, accumulating evidence suggests the presence of a “brain-first” and a “body-first” subtype of PD [22]. These subtypes are first proposed by Horsager and colleagues [23], but have not been supported by solid evidences to date [24]. Based on the brain-first subtype, the pathogenesis originates in one hemisphere and spreads across the brain. Post-mortem studies suggest that in most of these cases the pathogenesis likely begins in the olfactory bulb or amygdala [2, 5, 25]. In the body-first subtype, the pathogenesis originates from the peripheral nervous system, most likely within the enteric nervous system. It then spreads through autonomic nerves to the sympathetic trunk and brainstem, gradually ascending to affect the remainder of the brain. In support of a body-first phenotype, a post-mortem study reports that individuals with a caudo-rostral pattern of brain pathology (associated with the body-first profile) exhibit higher levels of spinal cord involvement compared to those with an amygdala-based pattern (brain-first profile) [26]. This study also finds that 23% of individuals with a body-first pattern do not exhibit olfactory bulb pathology, whereas all cases with a brain-first pattern do show olfactory bulb pathology [27]. Based on this, it can be suggested that these distinct subtypes differ phenotypically as well. Brain-first patients are expected to manifest a more asymmetric motor-predominant phenotype and later sleep and autonomic disorders. Body-first patients exhibit a longer period of gastric prodrome, early occurring REM sleep behavior disorder (RBD), and a higher burden of autonomic symptoms [22, 28]. The presence of pre-motor RBD seems to be a strong clinical indicator for differentiating between brain- and body-first subtypes. Dong et al. recently publish a study in which the PD group is subdivided into brain-first (post-RBD and non-RBD) and body-first (pre-RBD) subtypes [9]. They report a significant CSA reduction in the right and left vagus nerve in the body-first group compared to the brain-first group. The proposed atrophy of the vagus nerve may provide supporting evidence for the hypothesis of brain- and body-first subtypes.

### Autonomous symptoms and vagus nerve atrophy

The individual studies show diverse results; some reported no reduction in CSA in PD patients [10, 12, 15, 18], while others find a reduced CSA [8, 9, 11, 13, 14, 16, 19, 20]. An important factor that may explain differences between studies is the presence of autonomic symptoms. The relationship between autonomic symptoms in PD patients and the CSA of the vagus nerve is complex. Autonomic dysfunction may be related to changes in the vagus nerve, including axonal loss and demyelination, affecting the nerve’s ability to regulate autonomic nervous system functions. The severity of autonomic dysfunction can be assessed by the SCOPA-AUT, a validated questionnaire to assess autonomic dysfunction in PD [29]. Similar to other studies, in our cohort we do not find a correlation between the CSA of the vagus nerve and autonomic dysfunction [8, 10, 16, 18]. Also, in another disorder, namely type 2 diabetes, which is often associated with autonomic dysfunction, no correlation is found between the CSA of the vagus nerve and autonomic dysfunction [30]. Several other confounding factors may influence the relationship between vagus nerve CSA and autonomic dysfunction in PD, including disease severity, medication use, and comorbid conditions. Consequently, further research is essential to unravel the complex interplay between these variables and vagus nerve CSA in patients with PD. At present, it remains unclear whether observed differences in CSA are specifically related to PD pathology or reflect autonomic dysfunction more broadly. Future studies should measure autonomic symptoms in parallel and have sufficient power to observe differences in vagus nerve CSA in patients with and without autonomic dysfunction.

### Methodological considerations

Studies included show variation in CSA values, ranging from a mean CSA of 0.87 mm^2^ to 3.3 mm^2^ in the control groups. This discrepancy may be explained by differences in selection and recruitment, hardware used, transducer resolution, probe position, angle of insonation, and assessor experience [31, 32]. Similar variation is also observed in studies of vagus nerve ultrasound in other disorders [33, 34]. It is important to note that the CSA of the vagus nerve can be enlarged in inflammatory, autoimmune, or vasculitis-induced neuropathies [35]. When selecting participants, individuals with an enlarged CSA should therefore be excluded. Moreover, it should be noted that most studies did not separate ‘body first’ and ‘brain first’ phenotypes.

This meta-analysis shows a difference at the group level, but the inter-individual variation in CSA is large. Therefore, the clinical utility for diagnosis, that is, correctly classifying single patients, is limited despite statistical significance. Clinicians should not use vagus nerve ultrasound as a PD biomarker or diagnostic test in current practice.

## Conclusion

This meta-analysis reveals a reduction in vagus nerve CSA in patients with PD detectable at group level. Future studies are needed to clarify the role of vagus nerve ultrasound in monitoring disease progression and its relation to autonomic dysfunction.

## Supplementary Information

Below is the link to the electronic supplementary material.

Supplementary file1 (XLSX 13 KB)

## Data Availability

Data are available in Supplementary 1.
