# Correction: Ultrasonography demonstrates vagus nerve atrophy in Parkinson’s disease: a meta-analysis

**DOI:** 10.1007/s00415-026-14086-4

**Published:** 2026-09-16

**Authors:** Laura C. J. Sijben, Sander M. J. van Kuijk, Ali Jahanshahi, Werner H. Mess, Marcus L. F. Janssen

**Affiliations:** 1 Department of Clinical Neurophysiology, Maastricht University Medical Center, P. Debyelaan 25, 6229 HX Maastricht, the Netherlands; 2 Mental Health and Neuroscience Research Institute, Faculty of Health, Medicine and Life Sciences, Maastricht University, Universiteitssingel 40, 6229 ER Maastricht, the Netherlands; 3 Department of Neurology, Maastricht University Medical Center, P. Debyelaan 25, 6229 HX Maastricht, the Netherlands; 4 Department of Clinical Epidemiology and Medical Technology Assessment, Maastricht University Medical Center, P. Debyelaan 25, 6229 HX Maastricht, Netherlands; 5 Department of Neurosurgery, Maastricht University Medical Center, P. Debyelaan 25, 6229 HX Maastricht, the Netherlands

**Correction: Journal of Neurology (2026) 273:463** **https://doi.org/10.1007/s00415-026-13993-w**

In the original online version of this article, The Table 1 appeared incorrectly and have now been replaced by the corrected version.

**Table 1:**
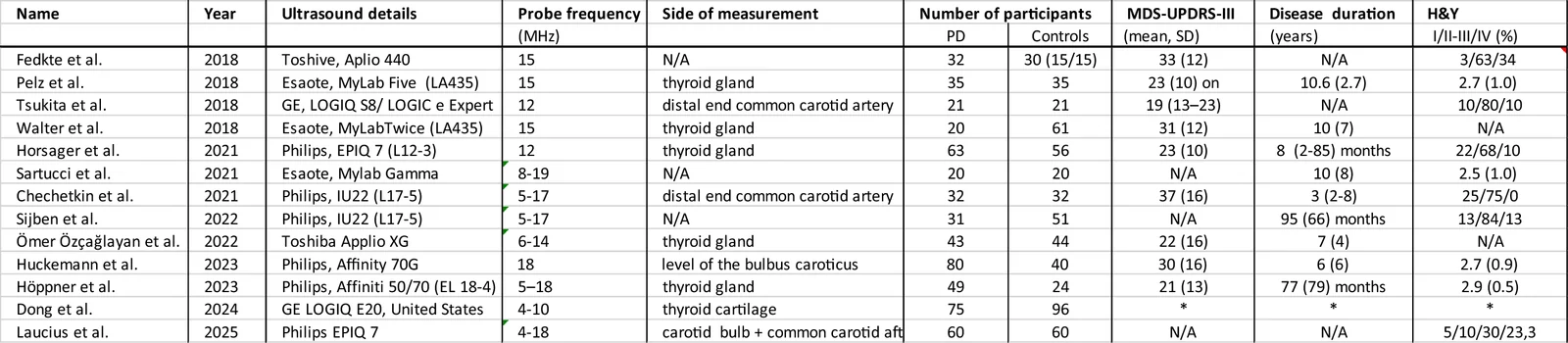
Details of the included studies are given, including year of publication, ultrasound device, side of measurement, number of participants (Parkinson’s disease and control), MDS-UPDRS III score, disease duration Hoehn and Yahr score.

Values are presented as means + SD

The original article has been corrected.

